# Analysis of genome-wide association studies of low-temperature germination in Xian and Geng rice

**DOI:** 10.3389/fpls.2024.1404879

**Published:** 2024-08-06

**Authors:** Kang Li, Muhammad Ahmad Hassan, Jinmeng Guo, Xueyu Zhao, Quan Gan, Cuixiang Lin, Bin Ten, Kunneng Zhou, Min Li, Yingyao Shi, Dahu Ni, Fengshun Song

**Affiliations:** ^1^ Rice Research Institute, Anhui Academy of Agricultural Sciences, Hefei, China; ^2^ College of Agronomy, Anhui Agricultural University, Hefei, China

**Keywords:** rice, LTG, GWAS, QTLs, low-temperature, breeding

## Abstract

Rice is the leading global staple crop. Low temperatures pose negative impacts on rice’s optimal growth and development. Rice cultivars acclimating to low temperatures exhibited improved seedling emergence under direct-seeded sowing conditions, yet little is known about the genes that regulate germination at low temperatures (LTG). In this research investigation, we’ve performed whole genome sequencing for the 273 rice plant materials. Using the best linear unbiased prediction (BLUP) values for each rice material, we identified 7 LTG-related traits and performed the efficient genetic analysis and genome-wide association study (GWAS). As a result of this, 95 quantitative trait loci (QTLs) and 1001 candidate genes associated with LTG in rice were identified. Haplotype analysis and functional annotation of the candidate genes resulted in the identification of three promising candidate genes (*LOC_Os08g30520* for regulating LTG4 and LTG5, *LOC_Os10g02625* for regulating LTG6, LTg7 and LTG8, and *LOC_Os12g31460* for regulating LTG7, LTg8 and LTG9) involving in the regulation of LTG in rice. This research provides a solid foundation for addressing the LTG issue in rice and will be valuable in future direct-seeded rice breeding programs.

## 1 Introduction

Rice is one of the major food crops in the world, ensuring food for more than 50% of the global population (Sreenivasulu et al., 2021). However, meeting the future dietary demands of a growing population is at risk due to uncertain climate variations (Hassan et al., 2023). In recent decades, there has been a notable increase in extreme temperature events (IPCC, 2014). Low temperature poses a significant challenge to the growth and development of rice plants, impacting their attributes, geographic distribution, and productivity (Sperotto et al., 2018). Rice originated in the tropics and subtropics and is more susceptible to the negative impacts of low-temperature stress than other grain crops such as wheat, barley, and soybean (Zhang et al., 2014; Kovach et al., 2007). As the world population increases and arable land diminishes, the rise in rice production poses a formidable challenge to food security and national economies. With the increasing demand for food, the rice planting area continues to expand from tropical and subtropical regions to high-altitude and high-latitude regions. The probability of rice suffering from chilling injury is gradually increasing. About 24 countries in the world (such as China, Japan, and North Korea, etc.) have encountered severe chilling injury (Zhang et al., 2017). In the double-cropping rice areas of South China and the middle and lower reaches of the Yangtze River, early-grown rice often suffered from cold stress (Liu et al., 2018). The difference between the north and south of the rice area in China is about 34 degrees (53°27’N in the northernmost Amur River and 18°90’N in the southernmost Hainan), and the difference in altitude is 2700 meters (from the southeast coast to the Yunnan-Guizhou Plateau). The changes in ambient temperature expose rice cultivation to various risks, particularly low-temperature threats, which annually lead to a substantial rice yield loss of 3~500 million tons of grain production in China (Zhu et al., 2015; Zhang et al., 2017). Therefore, low temperature is a significant constraint to global rice production (Shinada et al., 2013), as it usually results in reduced seedling survival, stunted growth, and decreased tillering during the active growth stages of rice, such as germination and seedling stage, which in turn directly affects the yield and quality of rice (Xu et al., 2020).

In recent years, direct-seeded rice has become increasingly popular worldwide due to the continuous reduction of labor and its low cost, easy planting, and quick management (Zhu et al., 2015). However, direct-seeding rice demands good low-temperature germination traits for adequate seedling survival. Environmental and genetic interactions influence rice’s low-temperature germination (LTG) (Yang et al., 2019). Approximately 300 quantitative trait loci (QTLs) have been identified across 12 rice chromosomes under low-temperature stress conditions through mapping, cloning, and SSR marker analysis in various populations such as RIL, NIL, BIL, DH, BC, and natural populations (Xu et al., 2008; Shirasawa et al., 2012; Jiang et al., 2017, 2020). It is reported that Jiang et al. (2017) successfully identified six LTG-related QTLs in 124 rice backcross recombinant self-compatible lines derived from a cross between the Xian rice variety Changhui 891 and the Geng rice variety 02428. Li et al. (2013) identified three LTG-regulating QTLs by constructing a RIL population and finely localized the largely contributing QTL (*qLTG-9*) with a 72.3 kb region in chromosome 9. Ji et al. (Ji et al., 2008) discovered 11 QTLs through the QTL localization of rice varieties in three environments; two QTLs had a maximum phenotypic variance explained (PVE) percentage of 27.9%. Cao et al. (2022) detected two QTLs (*qLTG-3* and *qLTG-12*) on chromosomes 3 and 12, highlighting a mutual effect that increased germination rates by 22-27%. Yang et al. (2018) succeeded in localizing 12 QTLs related to LTG in a backcross population from the cross between Dongnong422 and Kongyu131. Among the identified QTLs, only *qLTG3-1* was successfully cloned. During seed germination, *qLTG3-1* is particularly characterized by its expression in the seed coat’s aleurone layer and the germ sheath’s epiblast covering (Wang et al., 2023). It improves seed germination at low temperatures by regulating cell vacuolization in these tissues, leading to enhanced plasticity in tissues that reduces the mechanical resistance to the growth of the germinal sheath (Fujino and Matsuda, 2010; Wang et al., 2023).

Finding LTG-related loci in rice has been effective when candidate genes for LTG are analyzed using high-density single nucleotide polymorphisms (SNPs) and statistical modeling (Fujino et al., 2015; Mao et al., 2022; Li et al., 2022a). Li et al. (2022a) successfully identified 18 QTLs by integrating six LTG-related indicators with genome-wide association studies (GWAS) in a natural population of 211 rice lines. Mao et al. (2022) identified 37 QTLs using 497k SNPs. Wang et al. (2018b) discovered 53 QTLs through GWAS and also identified the gene (*OsSAP16*) responsible for regulating LTG in rice. They found that increased expression of *OsSAP16* enhances germination at low temperatures and vice versa. However, for a better understanding of the genetic basis of LTG in rice, more LTG-related genes must be excavated using GWAS, as fewer genes have been discovered concerning the regulation of LTG in rice.

Plants have developed complex regulatory networks to adapt to different temperature conditions (Li et al., 2022a). Rice cultivation in Asia is divided into two subspecies, *indica* (Xian) and *japonica* (Geng), and each subspecies contains many materials with different levels of low-temperature tolerance (Ma et al., 2015). Utilizing these rich genetic resources to identify numerous low-temperature tolerance-related genes and loci in rice has become a high priority. To analyze the LTG rate for 3 to 9 days, we assembled a natural population comprising 273 rice samples and acquired approximately 4.93 million SNPs through resequencing. This research investigation enabled us to explore the underlying genetics of LTG in rice. We opted for the best linear unbiased prediction (BLUP) values for seven traits across two different environments and locations to mitigate environmental variability and diminish false positives. We evaluated each trait individually and successfully identified 95 QTLs and three potential candidate genes. The findings of our research investigation serve as a fundamental groundwork for gaining a more profound understanding of the mechanisms governing LTG in rice.

## 2 Materials and methods

### 2.1 Plant materials

Plant material from 273 varieties/lines was procured from 25 provinces in China and six other countries, with 150 being Xian and 123 Geng rice varieties (**Supplementary Table S4**). It was planted in Shucheng and Hefei (Anhui, China) in 2022 and 2023, respectively. Each rice variety/line was sown with twenty plants in two rows containing ten plants per row and a row-to-row spacing of 25 cm. Two rows were planted at each border with 25 cm spacing between them to minimize border effects. The remaining practices, such as irrigation, fertilization, and pest control, were followed according to local agronomic standards. Upon reaching maturity (approximately 40 days after flowering), five randomly selected plants from each planting unit were harvested and utilized as test samples.

### 2.2 LTG measurement

The harvested seeds were naturally air-dried for two weeks and subsequently subjected to a 50°C oven treatment for seven days to break dormancy. This seven-day dry heat treatment is an easy and cost-effective method for breaking seed dormancy in rice plants (Shiratsuchi et al., 2017; Baskin and Baskin, 2020). The experiment was conducted with three replications, and thirty thoroughly dried and disease-free seeds were chosen for each replication. These seeds were sterilized by soaking them in 1.5% sodium hypochlorite for 30 minutes and then rinsed 4-5 times with distilled water. The seeds were placed in 9 cm petri plates containing two layers of filter paper and 10 ml of pure water, which was added through a pipette gun. Petri plates were then positioned in an artificial climatic chamber set at a photoperiod of 24-hour darkness, a temperature of 15°C, and 70% relative humidity. Seeds with a shoot or root length of more than 0.1 cm were defined as germinated seeds, and the number of germinated seeds was counted daily from day 3 until day 9 (Wang et al., 2018a; Wang et al., 2018c; Najeeb et al., 2020). The number of germinated seeds was counted on the 9^th^ day, then promptly moved to an artificial growth chamber, maintained at 30°C for an additional three days. Dead or non-germinating seeds that could not break dormancy were eliminated and disposed of. The number of germinated seeds was recorded after three days. The seed germination percentage was calculated as follows:


$$\begin{array}{c} \text{LT Seed germination }\%{\text{ on each day}}_{\left(\text{n}\right)}\\ =\;({\text{Germinated seeds on each day}}_{\left(\text{n}\right)}/\\ \text{ Number of seeds germinated at }\\ 30{}^{\circ}\text{C after }12\text{ days of sowing })*100 \end{array}$$

$$\text{Here},{\text{ day}}_{\left(\text{n}\right)}=\;3,\;4,\;5,\;6,\;7,\;8,\;9\text{ days}$$

Later, LTG was evaluated based on the germination percentage of each day.

### 2.3 Statistical analysis

Statistical data analysis was performed using Excel 2021, while the ‘R software’ was employed for daily correlation and frequency analyses of LTGs. The BLUP of each genotype-environment combination and variance component was obtained using the R package ‘Phenotype’ (Piepho et al., 2007). Furthermore, the R packages “corrploof” and “ggplot2” were used to generate correlation analysis plots and box plots depicting the phenotypic data.

### 2.4 DNA extraction and SNP genotyping

In each of the 273 plant samples prepared for sequencing, two leaves were harvested at the tillering stage (one month after seedling transplantation). Genomic DNA extraction was carried out using the standard CTAB method. Subsequently, libraries were prepared following the manufacturer’s instructions for sequencing on the MGI-DNBSeq platform, and the raw sequences were then subjected to further processing to eliminate low-quality reads containing adapters. The Beijing Genomics Institution was responsible for constructing libraries, sequencing, and sequence cleanup. The sequencing data were aligned to the reference genome (IRGSP-1.0) using the Burrows-Wheeler alignment (BWA) tool (Li and Durbin, 2009), and SNPs were called by employing the Genome Analysis Tool Kit (GATK) (DePristo et al., 2011). The SNPs with a minor allele frequency (MAF) of 5% and a deletion rate of ≤20% were retained using a whole genome association analysis toolset known as PLINK (Chang et al., 2015). Missing genotypes were inferred using a genotype imputation and haplotype phasing program IMPUTE2 (Howie et al., 2012), resulting in 4938656 high-quality SNPs. Density maps were plotted using the “Circle Manhattan Plot (CMplot)” software package (Yin et al., 2021) to show the distribution of variants in the 12 chromosomes.

### 2.5 Population structure analysis

The population structure was analyzed using principal component analysis (PCA) and neighbor-joining (NJ) tree techniques. Genetic distances were calculated using TASSEL (Trait analysis by Association, Evolution, and Linkage) (Bradbury et al., 2007), and the NJ tree was visualized with the online tool iTOL (interactive tree of life) (https://itol.embl.de/). PCA was performed using PLINK software and plotted using the R package ‘ggplot2’.

### 2.6 Genome-wide association studies

This study obtained 4938676 SNPs (MAF > 0.05) and 7 phenotypic data sets. These SNPs and phenotypic data were utilized for Genome-Wide Association Study (GWAS) analysis in Tassel software employing a general linear model (GLM) (Price et al., 2006). The Bonferroni correction controlled genetically false positives in this population to obtain a threshold p-value (p = 2.02e-9) for GLM. Significant SNPs were those with a p-value lower than the established threshold. SNPs in the same linkage disequilibrium (LD) region were the same QTL. Here, the LD attenuation distance was 200 kb, following earlier reports of LD attenuation distances of 100–200 kb in cultivated rice (Huang et al., 2010). The SNPs with the lowest p-value were used as the leading SNPs, and the neighboring SNPs within the physical distance of 200 kb were merged into the same QTL; the Manhattan plot was drawn with the software R package “CMplot.”

### 2.7 Candidate gene identification and haplotype analysis

To identify candidate genes associated with low-temperature germination (LTG), the Rice Genome Annotation Project (http://rice.plantbiology.msu.edu) was used for the screening of specific SNPs linked to the potential candidate genes and located in the 200 kb genomic area. We removed retrotransposons, transposons, genes encoding, and hypothetical proteins from the pool of candidate genes. Subsequently, R software was utilized to extract and evaluate SNP data for the candidate genes for haplotypes >10, and a t-test was employed to assess whether or not the locus affected LTG in rice.

## 3 Results

### 3.1 Phenotypic variation and correlation

**Figure 1:**
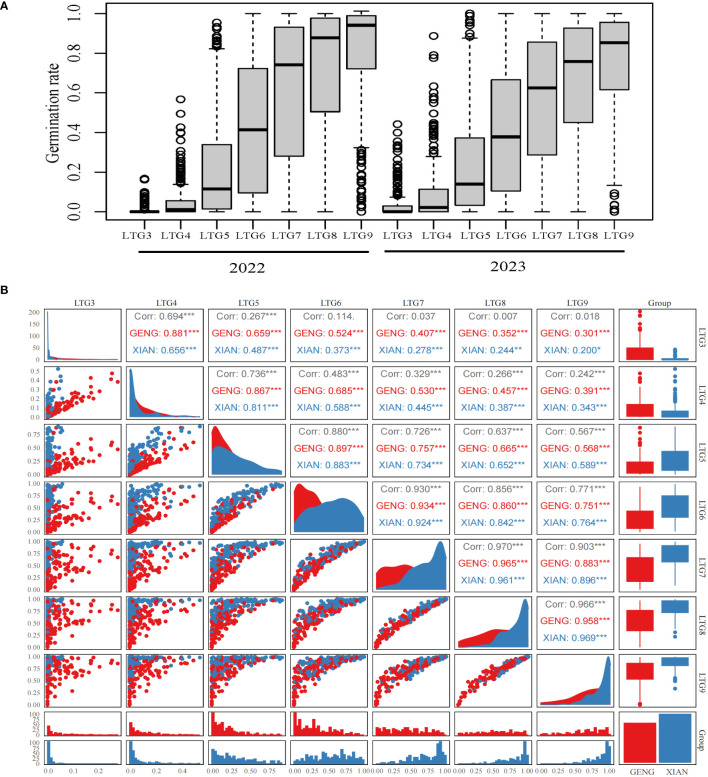
Description of LTG: **(A)** Germination rates in 2022 and 2023, **(B)** Correlation between different LTGs. Here, *, **, *** correlations are significant (p< 0.05, p< 0.01, p< 0.001).

To identify novel genes associated with LTG, 273 rice plant materials were evaluated for seven distinct LTG traits (LTG3, LTG4, LTG5, LTG6, LTG7, LTG8, and LTG9). The distribution of LTG traits assessed in 2022 and 2023 exhibited consistent trends; the average values from the two years were used for analysis, with the mean of the seven LTG traits being 0.0215, 0.0723, 0.2289, 0.4195, 0.5949, 0.6998, and 0.7825, respectively (refer to **Figure 1A**, **Table 1**). The plant materials from sample 273 were divided into Xian and Geng categories for comparison. It was observed that the average LTG rate in Geng was significantly higher than that of Xian on days 3 and 4, and the LTG rate in Xian started exceeding that of Geng from day 5 onwards. Correlation analysis indicated significant relationships among all traits except LTG3. A separate comparative analysis of the Xian and Geng varieties also revealed significant correlations among all characteristics (refer to **Figure 1B**). These results indicated that rice LTG has a substantial genetic variation.

**Table 1:** Description of germination rate in the full population.

Trait	Mean ± SD	Range	Median
LTG3	0.0215 ± 0.0442	0-0.2710	0
LTG4	0.0723 ± 0.1010	0-0.5230	0.0255
LTG5	0.2289 ± 0.2192	0-0.8985	0.1762
LTG6	0.4195 ± 0.2830	0-0.9890	0.3990
LTG7	0.5949 ± 0.2905	0-1	0.6392
LTG8	0.6998 ± 0.2610	0-1	0.7625
LTG9	0.7825 ± 0.2201	0-1	0.8582

### 3.2 SNP density analysis and population structure analysis

**Figure 2:**
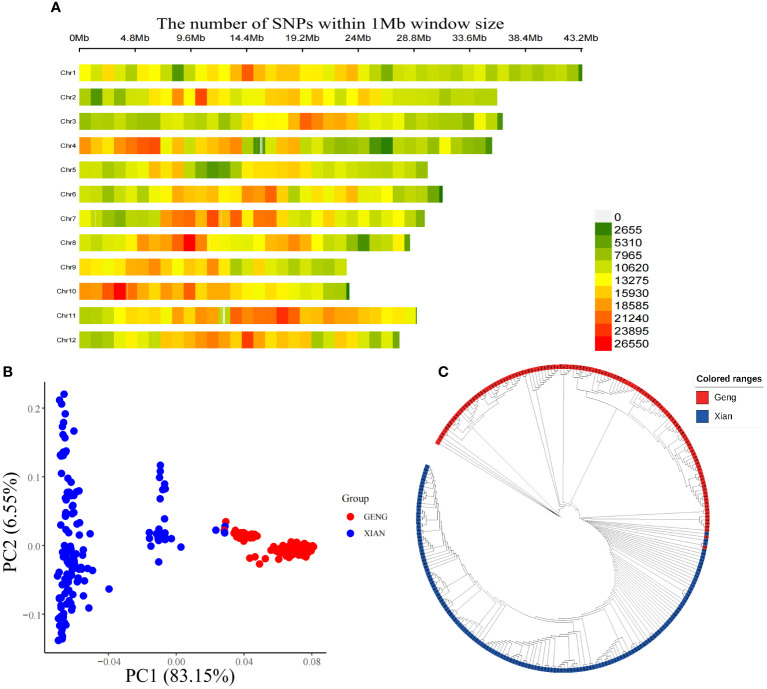
Genetic evolution of populations. **(A)** SNP density map, **(B)** Principal component analysis and **(C)** Neighbor-joining (NJ) tree.

After contrasting the sequencing data to the reference genome, 12.64 million SNPs were obtained. There were 4938676 SNPs obtained after filtering, which were evenly distributed on 12 chromosomes, and the average density of the 12 chromosomes was 13,231.71/Mb. Thus, the SNPs used for GWAS are sufficient for investigation (**Figure 2A**; **Supplementary Table S1**). The principal component analysis (PCA) and NJ tree were employed to analyze the population structure of the 273 plant materials. The total variance explained by principal component-1 (PC1) and principal component-2 (PC2) in PCA was 83.15% and 6.55%, respectively, and the NJ tree was plotted according to the genetic distances of each material. The PCA and NJ tree results showed that the studied population was divided into two subgroups corresponding to the Xian and Geng rice, which was in line with the subdivision of the test material consistent with the clustering of the experimental materials (**Figures 2B, C**).

### 3.3 GWAS for LTG in rice

**Figure 3:**
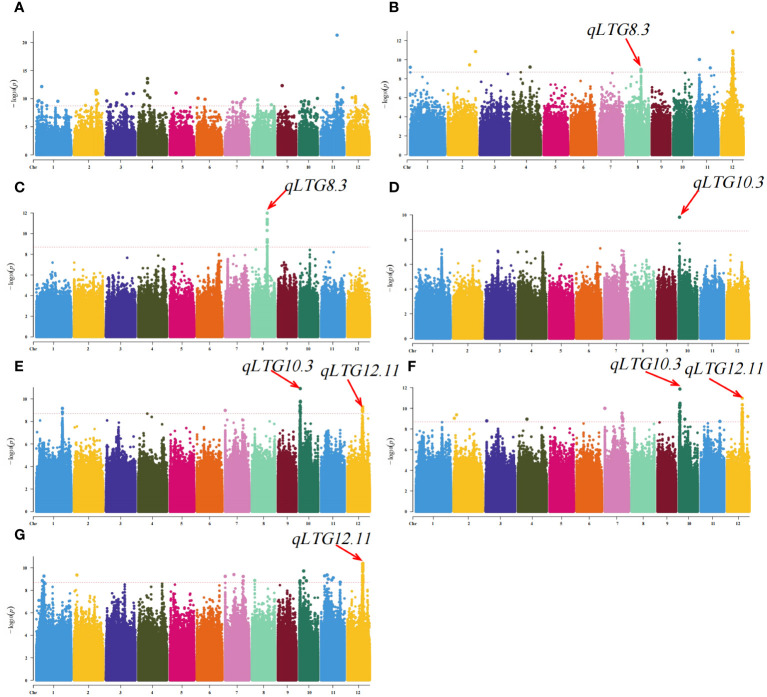
Genome-wide association analysis of rice LTG using GLM. **(A)** LTG3, **(B)** LTG4, **(C)** LTG5, **(D)** LTG6, **(E)** LTG7, **(F)** LTG8 and **(G)** LTG9.

In this study, from the genome sequences of 273 rice materials, a grand total of 4,938,656 high-quality SNPs were discovered, along with the collection of phenotypic data for seven LTG traits (LTG3, LTG4, LTG5, LTG6, LTG7, LTG8, and LTG9). The phenotypic and SNP data were analyzed using the GLM model GWAS. A total of 292 significant SNP loci associated with LTG were identified using an association threshold of p = 2.02e-9. (**Figure 3**; **Supplementary Table S2**). Considering the decay distance of rice LD, adjacent SNPs within a range of less than 200 kb were categorized as single QTLs, and the SNPs with the lowest p-values were denoted as the leading SNPs. This study successfully identified 13 previously reported QTLs and 82 novel QTLs, and 1001 candidate genes were obtained (**Table 2**; **Supplementary Table S3**). Among these, 54 QTLs were localized to LTG3, distributed across 12 chromosomes, with phenotypic contributions ranging from 10.58~26.11%, with the maximum phenotypic contribution of *qLG11.9* reaching 26.11%. For LTG4, a single QTL was found on chromosomes 1, 4, and 8, 2 QTLs on chromosomes 2 and 11, and 6 on chromosome 12. The phenotypic coefficient of variation of *qLTG12.7* was the highest on chromosome 12 (19.47%), and *qLTG12.4* had the smallest coefficient of variation on chromosome 12 (13.77%). Among these, *qLTG8.3* was 1 QTL co-localized on LTG5 and LTG4, with a phenotypic variation of 16.27%. LTG6 only localized 1 QTL (*qLTG10.3*) on chromosome 10, co-localized on LTG6, LTG7 and LTG8, with a phenotypic variation of 13.57%. LTG7 detected 1 QTL on chromosomes 1 and 7, and 2 QTLs on chromosomes 10 and 12; with phenotypic coefficients of variation of 10.90%, 11.74%, 11.96%, 13.57%, 10.99%, and 12.72%, respectively. LTG8 detected 2 QTLs on chromosome 2, 1 QTL on chromosomes 3, 4, and 11, and 3 QTLs on chromosomes 7 and 4 QTLs were detected on chromosomes 10 and 12 each, with the largest coefficient of variation for *qLTG10.3* (13.57%) and the smallest coefficient of variation for *qLTG3.2* (10.31%) on chromosome 3. LTG9 alone was detected on chromosomes 1, 7, 10, and 11, with 2, 1, 3, and 5 QTLs on each, respectively, and the coefficients of variation for the phenotypes ranged from 11.3% to 12.3%. 7 QTLs were detected as co-localized with other phenotypes. Among which *qLTG2.2*, *qLTG7.7*, *qLTG11.12*, and *qLTG12.12* were detected on both LTG9 and LTG8, with phenotypic variability of 11.87%, 11.72%, 11.13%, and 12.82%, respectively, and *qLTG7.1*, *qLTG10.2*, and *qLTG12.11* were repeatedly detected between the three phenotypes LTG7, LTG8 and LTG9 with 11.74%, 11.96%, and 12.72% phenotypic variation, respectively.

**Table 2:** Summary of detected QTLs using GLM in the full population.

QTLs	Trait	Chr	Pos	P	R² (%)	Previous QTLs/Genes
*qLTG1.1*	LTG4	1	184547	6.26E-10	14.34%	
*qLTG1.2*	LTG3	1	1222839	1.35E-09	12.05%	
*qLTG1.3*	LTG3	1	2090800	2.85E-10	12.89%	
*qLTG1.4*	LTG3	1	3289042	2.20E-10	13.03%	
*qLTG1.5*	LTG3	1	6737873	7.02E-13	16.06%	
*qLTG1.6*	LTG3	1	6956491	5.29E-10	12.56%	
*qLTG1.7*	LTG9	1	7503387	1.40E-09	11.29%	
*qLTG1.8*	LTG9	1	9497829	5.50E-10	11.76%	
*qLTG1.9*	LTG3	1	13093731	1.73E-09	11.91%	
*qLTG1.10*	LTG3	1	26775538	2.87E-10	12.89%	
*qLTG1.11*	LTG7	1	32405467	7.06E-10	10.90%	
*qLTG2.1*	LTG8	2	698792	9.24E-10	10.58%	
*qLTG2.2*	LTG8, LTG9	2	3469634	4.41E-10	11.87%	*qSR2-1* (Li et al., 2022b)
*qLTG2.3*	LTG3	2	14182567	1.56E-09	11.97%	
*qLTG2.4*	LTG4	2	26836681	3.51E-10	14.70%	
*qLTG2.5*	LTG3	2	27436799	6.02E-12	14.95%	
*qLTG2.6*	LTG3	2	27984356	1.76E-10	13.15%	
*qLTG2.7*	LTG3	2	29266551	1.06E-11	14.65%	
*qLTG2.8*	LTG3	2	29854412	7.52E-10	12.37%	
*qLTG2.9*	LTG4	2	34316505	1.41E-11	16.68%	
*qLTG3.1*	LTG3	3	1056363	2.46E-10	12.97%	
*qLTG3.2*	LTG8	3	1299595	1.65E-09	10.31%	
*qLTG3.3*	LTG3	3	5727116	1.16E-09	12.13%	
*qLTG3.4*	LTG3	3	12711085	1.70E-09	11.92%	
*qLTG3.5*	LTG3	3	13352563	5.37E-10	12.55%	
*qLTG3.6*	LTG3	3	20108228	1.35E-09	12.05%	
*qLTG3.7*	LTG3	3	25876386	1.48E-11	14.47%	*qLTG3c* (Fujino et al., 2015)
*qLTG3.8*	LTG3	3	34097328	1.15E-11	14.61%	*SNAC1; OsNAC9* (Hu et al., 2006)
*qLTG4.1*	LTG3	4	7067689	1.23E-09	12.10%	
*qLTG4.2*	LTG3	4	8026393	4.19E-12	15.14%	
*qLTG4.3*	LTG8	4	11134669	1.12E-09	10.49%	
*qLTG4.4*	LTG3	4	11551156	2.68E-14	17.73%	*qSR4* (Fujino et al., 2015)
*qLTG4.5*	LTG3	4	13508486	1.76E-09	10.76%	*qLTSS4-1* (Ma et al., 2015)
*qLTG4.6*	LTG3	4	14006650	1.88E-09	11.87%	
*qLTG4.7*	LTG3	4	14440441	6.54E-11	13.68%	
*qLTG4.8*	LTG4	4	22167803	5.97E-10	14.37%	
*qLTG5.1*	LTG3	5	7742011	9.49E-12	14.71%	*OsPRP* (Najeeb et al., 2020)
*qLTG6.1*	LTG3	6	1589915	8.36E-11	13.55%	*qLTG6a* (Fujino et al., 2015)
*qLTG6.2*	LTG3	6	10161883	1.29E-10	13.32%	
*qLTG7.1*	LTG7, LTG8, LTG9	7	54486	5.79E-10	11.74%	
*qLTG7.2*	LTG3	7	9661409	4.08E-10	12.70%	
*qLTG7.3*	LTG9	7	10880261	3.98E-10	11.93%	
*qLTG7.4*	LTG3	7	14692379	4.94E-10	12.60%	*qLTG7-5* (Shirasawa et al., 2012)
*qLTG7.5*	LTG3	7	19531660	6.81E-10	12.42%	
*qLTG7.6*	LTG3, LTG8	7	21460943	9.38E-10	10.58%	
*qLTG7.7*	LTG8, LTG9	7	22364235	5.92E-10	11.72%	
*qLTG7.8*	LTG3	7	24560676	1.06E-10	13.42%	
*qLTG8.1*	LTG9	8	3323895	1.34E-09	11.31%	
*qLTG8.2*	LTG3	8	7264027	1.79E-10	13.14%	
*qLTG8.3*	LTG4, LTG5	8	18832150	7.90E-12	16.27%	
*qLTG8.4*	LTG3	8	24347126	1.18E-09	12.12%	
*qLTG9.1*	LTG3	9	5579286	4.79E-13	16.26%	
*qLTG10.1*	LTG8, LTG9	10	439045	3.64E-10	11.02%	
*qLTG10.2*	LTG7, LTG8	10	719338	4.82E-11	11.96%	
*qLTG10.3*	LTG6, LTG7, LTG8	10	1007428	1.38E-12	13.57%	
*qLTG10.4*	LTG3	10	2602621	3.49E-10	12.78%	
*qLTG10.5*	LTG9	10	5237726	1.92E-10	12.29%	
*qLTG10.6*	LTG3	10	5526869	1.85E-09	11.88%	
*qLTG10.7*	LTG9	10	5750372	7.58E-10	11.60%	
*qLTG10.8*	LTG3	10	6830235	2.39E-10	12.99%	
*qLTG10.9*	LTG8	10	7106752	1.13E-09	10.49%	
*qLTG10.10*	LTG9	10	8543792	1.43E-09	11.27%	
*qLTG10.11*	LTG3	10	11747432	9.24E-10	12.26%	*qCTGERM10.11* (Wang et al., 2018c)
*qLTG10.12*	LTG3	10	22732244	9.01E-11	13.51%	
*qLTG11.1*	LTG9	11	4308453	5.25E-10	11.78%	
*qLTG11.2*	LTG4	11	5526788	9.62E-11	15.50%	*qCTGERM11-2/qLTSS11-1* (Schlappi et al., 2017) (Shakiba et al., 2017) [30,33]
*qLTG11.3*	LTG9	11	7425398	4.41E-10	11.87%	
*qLTG11.4*	LTG9	11	8844646	1.00E-09	11.46%	
*qLTG11.5*	LTG9	11	13117504	1.22E-09	11.35%	
*qLTG11.6*	LTG9	11	15084666	7.63E-10	11.59%	
*qLTG11.7*	LTG4	11	19011922	7.25E-10	14.25%	
*qLTG11.8*	LTG3	11	19750675	4.00E-10	12.71%	*L112/qCTS11-6* (DePristo et al., 2011)
*qLTG11.9*	LTG3	11	20252734	5.10E-22	26.11%	
*qLTG11.10*	LTG3	11	20464509	4.63E-11	13.87%	
*qLTG11.11*	LTG3	11	22589803	5.97E-10	12.49%	
*qLTG11.12*	LTG8, LTG9	11	23704763	1.88E-09	11.13%	
*qLTG11.13*	LTG3	11	25036230	1.10E-09	12.16%	
*qLTG11.14*	LTG3	11	25490114	1.66E-10	13.18%	
*qLTG11.15*	LTG3	11	25991253	4.09E-10	12.70%	*qLTGS(III)11* (Chang et al., 2015)
*qLTG11.16*	LTG3	11	27146452	4.11E-10	12.70%	
*qLTG11.17*	LTG3	11	27641914	1.15E-12	15.81%	
*qLTG12.1*	LTG3	12	6158274	6.40E-11	13.70%	
*qLTG12.2*	LTG3	12	10402588	8.26E-11	13.56%	
*qLTG12.3*	LTG3	12	10644373	1.25E-10	13.34%	*qBMSI12* (Chang et al., 2015)
*qLTG12.4*	LTG4	12	12953299	1.56E-09	13.77%	
*qLTG12.5*	LTG4	12	13438221	8.34E-10	14.16%	
*qLTG12.6*	LTG4	12	13944837	8.70E-10	14.14%	
*qLTG12.7*	LTG4	12	14148759	1.31E-13	19.47%	
*qLTG12.8*	LTG4	12	14701675	2.32E-11	16.38%	
*qLTG12.9*	LTG4	12	15015276	9.85E-10	14.06%	
*qLTG12.10*	LTG7, LTG8	12	18470876	3.91E-10	10.99%	
*qLTG12.11*	LTG7, LTG8, LTG9	12	18879706	8.32E-11	12.72%	
*qLTG12.12*	LTG8, LTG9	12	19139526	6.78E-11	12.82%	
*qLTG12.13*	LTG3	12	21983596	1.44E-09	12.02%	
*qLTG12.14*	LTG8	12	25714436	6.15E-10	10.77%	

### 3.4 Identification of candidate genes and haplotype analysis

#### 3.4.1 Identification of LTG4 and LTG5 candidate genes

**Figure 4:**
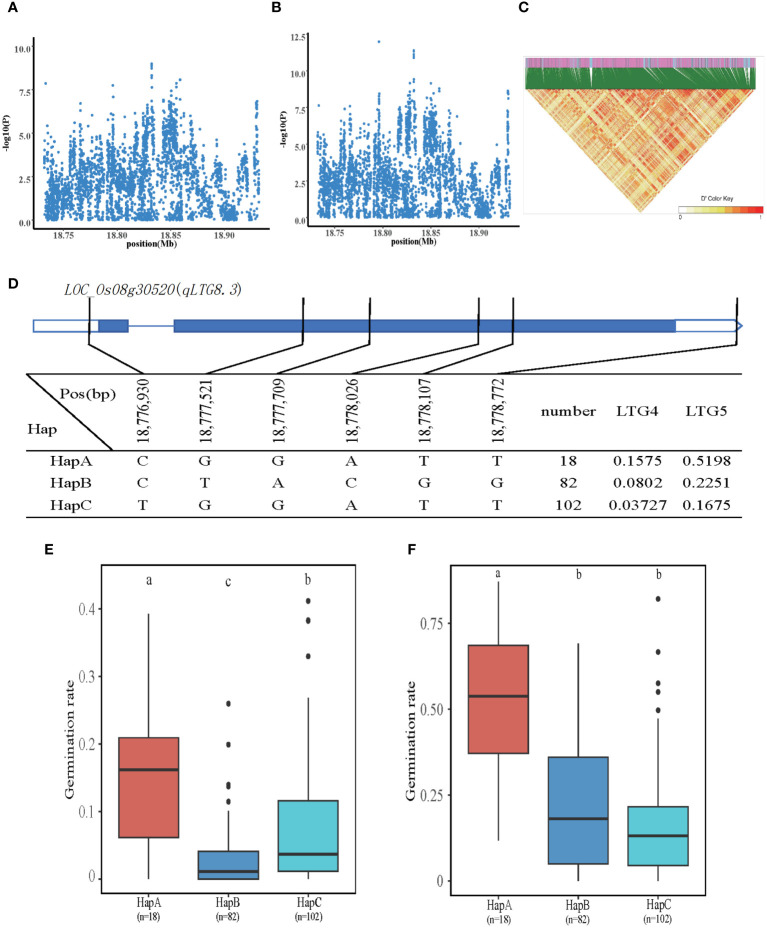
Identification of LTG4 and LTG5 candidate genes. **(A, B)** Local Manhattan plot (top) for LTG4 and LTG5. **(C)** The linkage disequilibrium heatmap for the region from 18.7Mb to 18.9Mb on chromosome 8. **(D)** Gene structure and haplotype analysis of *LOC_Os08g30520* based on SNPs from all evaluated rice varieties. Where thin blue lines indicate introns and intergenic regions, blue boxes and white boxes indicate exons and promoters, respectively, and thin black lines indicate the physical location of SNPs on the genome. Haplotypes of less than 10 rice varieties will not be counted. **(E, F)** Haplotypes of *LOC_Os08g30520* were statistically analyzed for LTG4 and LTG5 using Tukey’s test, and box plots demonstrate the differences between them. ‘a’, ‘b,’ and ‘c’ are based on whether the t-test is significant between each other.

In total, 13 QTLs were localized to LTG4 and LTG5, of which qLTG8.3 is a QTL co-localized by LTG4 and LTG5 at chromosome 8. We explored the Nipponbare genome reference sequence (http://rice.plantbiology.msu.edu/) to find genes that might impact the LTG candidate genes. Once genes encoding hypothetical proteins, retrotransposons, and transposon proteins were eliminated, we made predictions about potential candidate genes inside the QTL region. Five genes were identified at chromosome 8, 18.7Mb-18.9Mb. The SNPs of five genes were subjected to association analysis and haplotype typing, among which the most significant gene was *LOC_Os08g30520*, which was annotated as plant protein of unknown function domain-containing protein, located 54 kb upstream of the significant QTL, with two SNPs on the promoter and four SNPs on the exons. *LOC_Os08g30520* was detected in all materials with three major haplotypes: CGGATT (HapA), CTACGG (HapB), and TGGATT (HapC). The HapA, HapB, and HapC had mean LTG4 of 0.1575, 0.0802, and 0.03727, respectively. The mean LTG5 was 0.5198, 0.22561, and 0.1675, respectively. The two phenotypic values of HapA were significantly higher than the other two haplotypes. Therefore, it is hypothesized that *LOC_Os08g30520* is involved in regulating LTG4 and LTG5 (**Figure 4**).

#### 3.4.2 Identification of LTG6、LTG7 and LTG8 candidate genes

**Figure 5:**
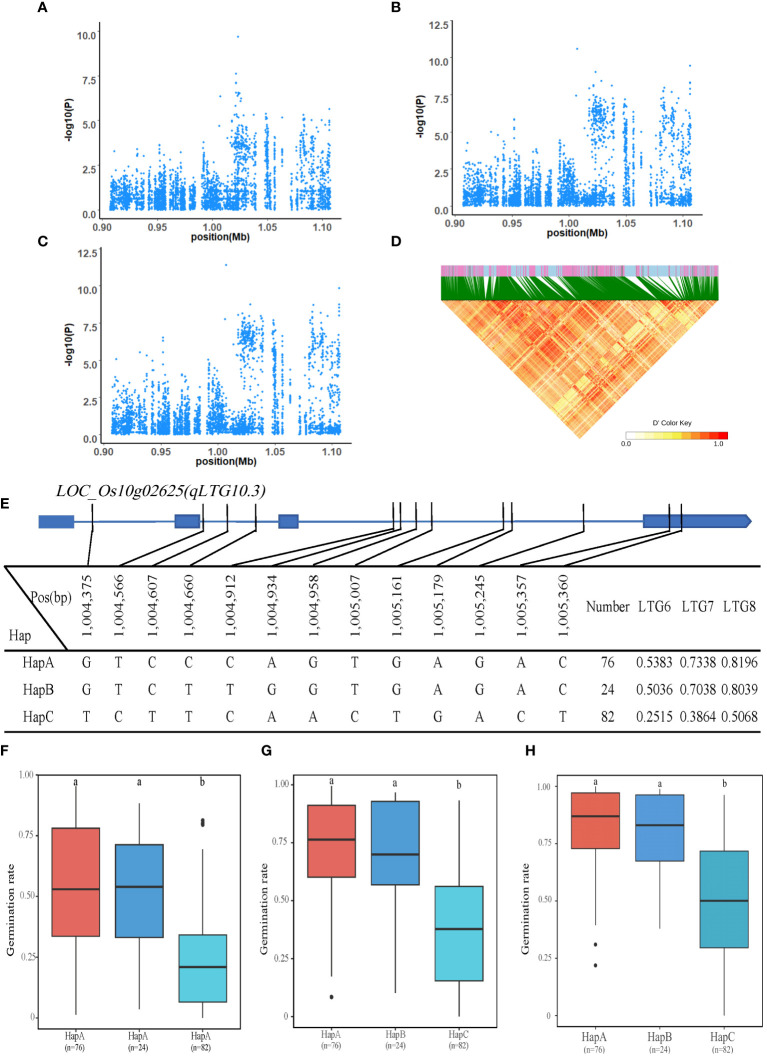
Identification of LTG6, LTG7 and LTG8 candidate genes. **(A–C)** Local Manhattan plot (top) for LTG6, LTG7 and LTG8. **(D)** The linkage disequilibrium heatmap for the region from 0.9Mb to 1.1Mb on chromosome 10. **(E)** Gene structure and haplotype analysis of *LOC_Os10g02625* based on SNPs from all evaluated rice varieties. Where thin blue lines indicate introns and intergenic regions, blue boxes and white boxes indicate exons and promoters, respectively, and thin black lines indicate the physical location of SNPs on the genome. Haplotypes of less than 10 rice varieties will not be counted. **(F-H)** Haplotypes of *LOC_Os10g02625* were statistically analyzed for LTG6, LTG7 and LTG8 using Tukey’s test, and box plots demonstrate their differences. ‘a’ and ‘b’ are based on whether the t-test is significant between each other.

In the interval of 0.9Mb-1.1Mb on chromosome 10 (*qLTG10.3*), 12 genes were identified. The *LOC_Os10g02625* was annotated as gibberellin-regulated protein, putative, expressed, which was located 3kb upstream of the significant QTL, with 11 SNPs on introns and 2 SNPs on exons. The *LOC_Os10g02625* has three major haplotypes such as HapA, HapB, and HapC, corresponding to GTCCCAGTGAGAC, GTCTTGGTGAGAC, and TCTTCAACTGACT, respectively. The mean LTG6 of HapA-C was 0.5383, 0.5036, and 0.2515; the mean LTG7 of HapA-C was 0.7338, 0.7038 and 0.3864; and the mean LTG8 of HapA-C was 0.8196, 0.8039 and 0.5068. Regarding LTG6, LTG7 and LTG8, HapC had the lowest and significantly lower values than the other two haplotypes (HapB and HapC). These results suggested that *LOC_Os10g02625* may be a candidate gene for LTG6, LTG7 and LTG8 (**Figure 5**).

#### 3.4.3 Identification of LTG7、LTG8、LTG9 candidate genes

**Figure 6:**
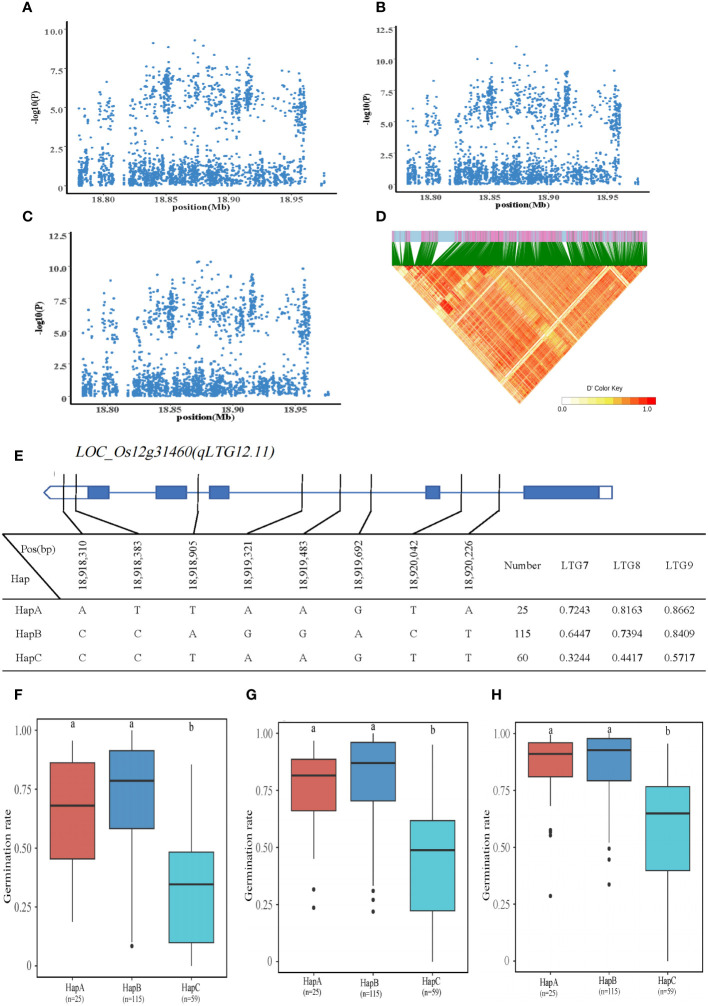
Identification of LTG7, LTG8 and LTG9 candidate genes. **(A–C)** Local Manhattan plot (top) for LTG7, LTG8 and LTG9. **(D)** The linkage disequilibrium heatmap for the region from 18.77Mb to 18.97Mb on chromosome 10. **(E)** Gene structure and haplotype analysis of *LOC_Os12g31460* based on SNPs from all evaluated rice varieties. Where thin blue lines indicate introns and intergenic regions, blue boxes and white boxes indicate exons and promoters, respectively, and thin black lines indicate the physical location of SNPs on the genome. Haplotypes of less than 10 rice varieties will not be counted. **(F-H)** Haplotypes of *LOC_Os12g31460* were statistically analyzed for LTG7, LTG8 and LTG9 using Tukey’s test, and box plots demonstrate their differences. ‘a’ and ‘b’ are based on whether the t-test is significant between each other.

This QTL, *qLTG12.11*, is co-localized on chromosome 12 and corresponds to LTG7, LTG8, and LTG9. A total of 10 genes were identified within the 18.77-18.97 Mb interval of chromosome 12. *LOC_Os12g31460* was annotated as a heat shock protein (HSP) located 41kb downstream of the significant QTL with two SNPs on the promoter and six SNPs on the intron. Three haplotypes, HapA is ATTAAGTA; HapB is CCAGGACT; and HapC is CCTAAGTT. The mean of LTG7 for HapA-C was 0.7243, 0.6447, and 0.3244, and the mean of LTG8 was 0.8163, 0.7394, and 0.4417; average LTG9 was 0.8662, 0.8409 and 0.5717. The HapC of LTG7, LTG8, and LTG9 was significantly lower than that of HapA and HapB. Therefore, it is anticipated that *LOC_Os12g31460* regulated LTG7, LTG8, and LTG9 in rice (**Figure 6**).

## 4 Discussion

Rice is a prominent food crop in China, and direct seeding of rice has significant development potential as a light, simple, and efficient cultivation practice (Devi, 2023). However, direct-seeded rice often faces low-temperature stress during germination, so exploring genes for rice varieties germinating at low temperatures is a vital step toward breeding programs regarding direct-seeded rice varieties. The key trait indexes for low-temperature tolerance in rice include the low-temperature germination rate and low-temperature germination potential (Ji et al., 2009; Wang et al., 2018b). The GWAS in plants is a method for identifying correlations between natural variation and phenotypic traits (Mackay and Powell, 2007). Through GWAS, researchers conduct whole genome sequencing or gene chip analysis on many individual plants and then analyze them using phenotypic data such as growth characteristics, yield, and resistance (Langridge et al., 2001). GWAS can help plant scientists find candidate genes or chromosomal regions (QTLs) associated with specific traits by comparing the associations between genotypes and phenotypic traits (Gutierrez et al., 2015). These findings are significant for resolving the genetic basis of plant traits, breeding improvement, and precision breeding. The application of GWAS methodology has provided researchers with a more comprehensive understanding of key traits and associated genes in plant genomes, thereby offering valuable insights for plant genetics and breeding studies.

In this research study, to minimize the impact of dormant seeds, we subjected freshly harvested rice seeds to dry heating at the temperature of 30°C to break dormancy. This process accelerated the physiological activity within the seeds, facilitating the breaking of dormancy and improving germination and growth rates. Furthermore, high-temperature drying aids in eliminating any bacteria and fungi present on the seed surface, thereby enhancing seed quality and storage stability. The low-temperature germination rate served as a metric to evaluate rice’s capacity to withstand low temperatures, with germination rates between 3 and 9 days in low-temperature settings serving as benchmarks. The findings indicated that Geng rice displayed higher LTG3 and LTG4 values under low temperatures compared to Xian rice, indicating a quicker germination pace in Geng rice during the initial 4 days. This result aligns with prior studies, including those by Morsy et al (Morsy et al., 2005). and Lv et al. (2016). Nevertheless, past the 5th day of the experiment, the low-temperature growth rate of Xian rice surpassed that of Geng rice, suggesting a potential gradual acclimatization of Xian rice to the low-temperature environment over time, corroborating previous suggestions during low-temperature treatments by Schlappi et al (Schlappi et al., 2017). These outcomes highlight the diverse responses of rice to low temperatures across different varieties and time frames, enhancing our comprehension of low-temperature tolerance mechanisms in rice. Furthermore, these findings offer valuable insights and references for future rice breeding programs that enhance low-temperature tolerance. Ongoing exploration of rice growth traits under varying temperature conditions enables the refinement of selection processes and breeding strategies for low-temperature-resistant varieties, ultimately boosting rice yield and stability.

In this study, 273 materials were genotyped, and 4938656 SNPs were used for genetic structure and GWAS analysis. 95 QTLs significantly associated with LTG were localized with a threshold value of -log10 (p) ≥ 8.6, and 1001 candidate genes were identified within the interval, indicating the genetic complexity of controlling low-temperature germination in rice. Combined with the haplotype analysis of the candidate genes, three important genes related to low-temperature germination in rice were screened. The *LOC_Os08g30520* refers to a type of protein that contains an unknown functional, structural domain in plants. Such proteins may regulate biological processes such as signaling, metabolic pathways, or gene expression by interacting with other proteins or biomolecules (Nietzsche et al., 2016). This kind of protein might be crucial for rice germination at low temperatures. Low temperature is an important environmental factor in the growth of rice and has a significant effect on its germination and growth processes. Therefore, plants’ adaptation to low temperatures through their physiological and molecular mechanisms is of prime importance. Proteins containing unknown functional, structural domains may regulate low-temperature signaling pathways, activate or repress specific gene expression, or regulate plant metabolic pathways, which affect rice growth and development under low-temperature adversity conditions. Therefore, investigating the contribution of such proteins to low-temperature adaptation in rice will help us better understand the mechanism of rice adaptation under low-temperature conditions. The protein encoded by gene *LOC_Os10g02625* is gibberellin-regulated. Gibberellin is a plant hormone that plays a vital role in plant growth and development (Castro-Camba et al., 2022). During low-temperature stress in rice, plants adjust their growth and development to adapt to these conditions. Gibberellin plays a regulatory role in low-temperature germination in rice (Yamauchi et al., 2004; Niu et al., 2014). Specifically, gibberellins promote seed germination and seedling growth, which is particularly important under low-temperature conditions and may inhibit plant growth (Yamauchi et al., 2004). Thus, the gibberellin signaling pathway, including gibberellin-regulated proteins, may significantly impact the adaptation of rice to low-temperature germination. *LOC_Os12g31460* is annotated as heat shock protein DnaJ, a class of molecular chaperone proteins belonging to the family of heat shock proteins. They play a crucial role in the physiological response of organisms subjected to environmental stresses such as high temperature, low temperature, oxidative stress, hypoxia, etc. (Tran Thi Ngoc et al., 2023). Further, DnaJ proteins are involved in the folding, assembly and depolymerization of proteins in the cell, helping other proteins to fold correctly and exhibit their functional conformation (Liu et al., 2021). It is also associated with many important cell signaling pathways, such as those that regulate the cell cycle, apoptosis and DNA repair, among other biological processes (Luo et al., 2023; Tran Thi Ngoc et al., 2023). In plants, the heat shock protein DnaJ has also been associated with environmental stresses, including low-temperature stress. Under low-temperature conditions, plants produce heat shock proteins to help them fight against adversity (Feder and Hofmann, 1999; Wang et al., 2005). Therefore, the heat shock protein DnaJ may be associated with low-temperature adaptation and cold tolerance in rice. Overall, heat shock protein DnaJ plays a vital role in plants’ response to environmental stress and survival under adverse conditions.

## 5 Conclusions

We performed an efficient genetic analysis of LTG in rice using natural population and genome-wide association analysis. A total of 95 QTLs associated with LTG in rice were identified (**Table 2**). After haplotype analysis and functional annotation of the candidate genes, three promising candidate genes (*LOC_Os08g30520*, *LOC_Os10g02625*, and *LOC_Os12g31460*) were successfully identified. This research investigation provides an essential basis for resolving the issue of LTG in rice, particularly in direct-seeded rice.

## Data availability statement

The original contributions presented in the study are publicly available. This data can be found at the National Center for Biotechnology Information (NCBI) using accession number PRJNA1141731 [URL: https://www.ncbi.nlm.nih.gov/bioproject/PRJNA1141731/].

## Author contributions

KL: Conceptualization, Data curation, Validation, Writing – original draft. MH: Conceptualization, Formal Analysis, Validation, Writing – original draft. JG: Data curation, Methodology, Writing – review & editing. XZ: Data curation, Software, Writing – original draft. QG: Data curation, Formal Analysis, Writing – review & editing. CL: Conceptualization, Methodology, Writing – original draft. BT: Software, Visualization, Writing – review & editing. KZ: Software, Validation, Writing – review & editing. ML: Software, Validation, Writing – review & editing. YS: Conceptualization, Methodology, Writing – original draft, Writing – review & editing. DN: Methodology, Supervision, Validation, Writing – original draft. FS: Investigation, Methodology, Supervision, Validation, Writing – original draft, Writing – review & editing.
